# 5-type HPV mRNA versus 14-type HPV DNA test: test performance, over-diagnosis and overtreatment in triage of women with minor cervical lesions

**DOI:** 10.1186/s12907-016-0032-x

**Published:** 2016-06-07

**Authors:** Bjørn Westre, Anita Giske, Hilde Guttormsen, Sveinung Wergeland Sørbye, Finn Egil Skjeldestad

**Affiliations:** Department of Pathology, Ålesund Hospital, Møre and Romsdal Health Trust, Ålesund, Norway; Department of Clinical Pathology, University Hospital of North Norway, 9038 Tromsø, Norway; Research Group Epidemiology of Chronic Diseases, Department of Community Medicine, UiT The Arctic University of Norway, Tromsø, Norway

**Keywords:** HPV, DNA, mRNA, Screening, Triage, CIN, CIN2, CIN3, Cervical cancer

## Abstract

**Background:**

Repeat cytology and HPV testing is used in triage of women with minor cytological lesions. The objective of this study was to evaluate 14-type HPV DNA and 5-type HPV mRNA testing in delayed triage of women with ASC-US/LSIL.

**Methods:**

We compared a DNA test (Roche Cobas 4800) and an 5-type mRNA test (PreTect HPV-Proofer). In total 564 women were included in the study.

**Results:**

The sensitivity among solved cases for CIN3+ were 100 % (15/15) for both tests. The sensitivity for CIN2+ of the HPV DNA test was 100 % (38/38) relative to 79 % (30/38) for the 5-type HPV mRNA test. The corresponding estimates of specificity for CIN2+ among solved cases were 84 % (393/466; 95 % CI: 81–88) and 91 % (451/498; 95 % CI: 88–93). The positive predictive values for CIN3+ were 13.5 % (15/111) for DNA+ and 19.5 % (15/77) for 5-type mRNA+. Significantly more women screened with 5-type mRNA than DNA returned to screening (81 % vs 71 %, *p <* 0.01). Subsequently, significantly fewer women were referred for colposcopy/biopsies/treatment (19 % (105/564) vs 29 % (165/564), *p <* 0.01).

**Conclusions:**

5-type HPV mRNA is more specific than 14-type HPV DNA in delayed triage of women with ASC-US/LSIL. The referral rate for colposcopy was 57 % higher for DNA+ relative to mRNA+ cases (165 vs 105), with the same detection rate of CIN3+, but the 5-type mRNA test had lower sensitivity for CIN2+. It is important to consider the trade-off between sensitivity and specificity of the diagnostic test when designing screening algorithms.

## Background

Cervical cancer is the third most common cancer in women worldwide [1]. Persistent infection of human papillomavirus (HPV) causes virtually all cases of cervical cancer [2]. In Europe most cervical cancer cases are caused by HPV types 16, 18, 31, 33, and 45 [1, 3]. Cervical cancer can be prevented by early detection and treatment of precancerous lesions [4]. Women with minor cytological cervical lesions have an increased risk of having, or developing, high-grade dysplasia compared to women with normal cytology. However, most minor cytological lesions regress spontaneously, and therefore careful triage is crucial in order to avoid unnecessary referrals and overtreatment [5]. In Norway, HPV test is used in delayed triage of women with atypical squamous cells of undetermined significance (ASC-US) or low-grade squamous intraepithelial lesions (LSIL) [6]. If the HPV test is positive, the woman is referred to colposcopy.

The HPV E6/E7 mRNA test PreTect HPV-Proofer which detects HPV E6/E7 mRNA from the five most prevalent types causing cervical cancer has been shown to have a higher clinical specificity and positive predictive value (PPV) than HPV DNA tests [7–14]. A high specificity and a low positivity rate of a triage test indicates a low referral rate for colposcopy [8]. In this study we performed a direct comparison of a 5-type HPV mRNA and a 14-type HPV DNA test in delayed triage of ASC-US/LSIL related to referral rates for colposcopy, biopsy rates, and histological outcomes.

## Methods

Organized cervical cancer screening was introduced in Norway in 1995 with the recommendation that all women 25 to 69 years have a Pap smear collected every third year [15]. During the study period the Norwegian cervical cancer program recommended delayed triage with repeat cytology and HPV testing 6–12 months after the index diagnosis of ASC-US/LSIL. Women with high-grade squamous intraepithelial lesions (HSIL) or repeated ASC-US/LSIL with a positive HPV test were referred to colposcopy/biopsy immediately after triage. Women with a normal smear and a positive HPV test were recommended a repeat HPV test within 12 months, whereas women with an ASC-US/LSIL/normal smear with a negative HPV test were returned to the screening program at a three-year interval [9].

This study compared test performance of the HPV mRNA test PreTect HPV-Proofer (PreTect AS, Norway), which detects E6/E7 mRNA of 5 HPV types, and the HPV DNA test Cobas 4800 (Roche Molecular Diagnostics), which detects 14 HPV types. We followed the manufacturer’s instructions in preparation of aliquots and detection of mRNA, while we analyzed HPV DNA in accordance with national guidelines [10]. The conventional cytology (Pap smear) consists of sampling cells from the cervical area. The sample is obtained using a brush, and the cells are placed directly onto a glass slide and spray fixed. Then the same brush is placed into a liquid medium (ThinPrep, Cytyc Corporation, Marlborough, USA) for HPV testing. In Norway, many hospitals have switched from conventional Pap smears to liquid-based cytology (LBC), but Ålesund Hospital still uses conventional Pap smears.

**Fig. 1:**
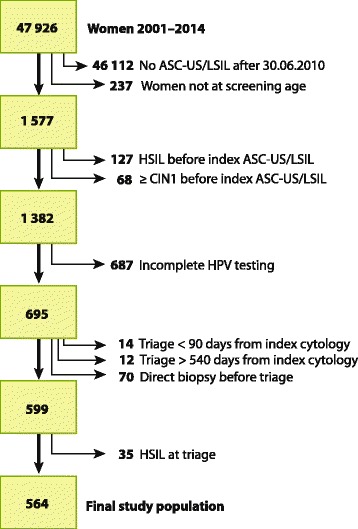
Selection of study population

The Department of Pathology, Ålesund Hospital, located on the western coast of Norway, serves a background population of approximately 50 000 women at screening age 25–69 years and assesses 12 000 cervical smears annually. Since 1999 the department has used the clinical database SymPathy for administration of cytological and histological specimens. From January 1, 2001, through September 15, 2014, we identified 47 926 women with 160 466 valid smears, among which 1 577 women had a diagnosis of ASC-US/LSIL after June 30, 2010. Our study commenced on January 4, 2012, when the department introduced the HPV DNA test. After excluding women with a history of HSIL, or biopsy with cervical intraepitelial neoplasi grade 1 or worse (CIN1+), those under 25 or over 69 years of age, and cases with none or only one HPV test, 695 women were eligible for study participation (Fig. 1).

**Fig. 2:**
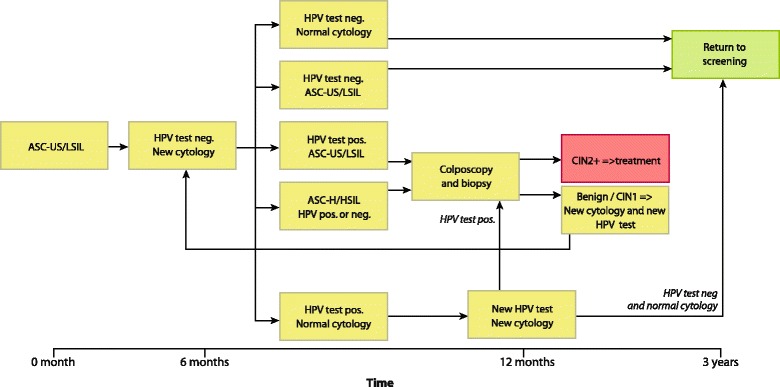
Screening algorithm for HPV triage

The Norwegian cervical cancer screening program recommended triage 6 to 12 months after the index ASC-US/LSIL [6, 9] (Fig. 2). We expanded the triage follow-up window from 90 to 540 days after the index smear. Therefore women having triage <90 days (*n =* 14) or >540 days (*n =* 12) after index smear, and women having direct biopsy (reflex testing) before or at triage (*n =* 70), and women who had HSIL at triage (*n =* 35) were excluded, leaving 564 women for final analyses. Either a positive HPV DNA or a positive HPV mRNA test triggered colposcopy.

We defined solved cases as subjects who returned to the screening program from either a valid smear/negative HPV test, or having had a biopsy, which determined future follow-up/treatment. Corresponding dates were “outcome” dates for solved cases, while we censored cases not met for biopsy or incomplete follow-up at last day of study, September 15, 2014. Abnormal cervical cytology was classified using the Bethesda system. Cervical biopsies were reported using WHO histological classification of tumors of the uterine cervix (http://screening.iarc.fr/colpochap.php?chap=2). All biopsies were reviewed by one experienced pathologist (BW). Biopsies with uncertain cellular changes were immunostained with p16 (INK4a) (Roche mtm laboratories AG). If there was a discrepancy between biopsy and treatment histology, the most severe histology was endpoint.

The sensitivity of the HPV tests is defined as the proportion of high-grade dysplasia (CIN2+) detected by the two different HPV tests. In the calculations of specificity, it is assumed that HPV negative samples without detected dysplasia during the follow-up period were disease-free.

All analyses were done in SPSS, version 22.0, with Chi-square test for categorical variables, *t*-test for continuous variables, and survival analyses for clinically solved cases. Significance level was set to *p <* 0.05.

## Results

At index cytology 84 % (473/564) were ASC-US and 16 % (91/564) LSIL. At the most recent screen prior to index cytology 79 % (444/564) of the women had a normal cytology within the screening interval, 4 % (24/564) had a normal cytology beyond the screening interval, whereas index cytology represented the first smear, ever, for 17 % (96/564) of the women. The mean age was 39 years (SD 10.5 years) and nearly 40 % (217/564) of the women were 25–34 years of age.

Mainly from triage, but also after follow-up of a normal cytology with a positive HPV test at triage, the 5-type mRNA test scheduled significantly more women back to screening, 81 % (459/564), than the DNA test, 71 % (399/564) (*p <* 0.01). There was no difference in incomplete follow-ups by screening test, HPV DNA test 4 % (24/564)/mRNA test 2 % (13/564) (*p =* 0.09). Accordingly, the DNA test targeted significantly more women for biopsy, 25 % (141/564) than the 5-type mRNA test, 16 % (92/564) (*p <* 0.01) (Table 1). In total 141 women were recommended colposcopy by the DNA test, and 105 (74 %) met for biopsy. Out of the 92 women scheduled for biopsy by the 5-type mRNA test, 77 (84 %) made a visit (*p =* 0.12).

**Table 1:** Outcome of triage by HPV test (*n =* 564)

	DNA		5-type-mRNA	
	*N =* 564	% (95 % CI)	*N =* 564	% (95 % CI)
Back to screening	399	70.7 (66.9–74.5)	459	81.4 (78.2–84.6)*
Met for biopsy	105	18.6 (15.4–21.8)	77	13.7 (10.9–16.5)
Scheduled, not met for biopsy	36	6.4 (4.4–8.4)	15	2.7 (1.4–4.0)*
Incomplete follow-up	24	4.3 (2.6–6.0)	13	2.3 (1.1–3.5)

*p < 0.05

*Triage* repeat cytology and HPV test 3–18 months after index ASC-US/LSIL cytology

*DNA* HPV DNA test (Cobas 4800)

*5-type-mRNA* HPV mRNA test (PreTect HPV-Proofer)

There was no difference in histology outcome by screening test among women who had biopsy and/or treatment. Both tests identified 14 women with CIN3 and one woman with squamous cell carcinoma (Table 2). The positive predictive value (PPV) for CIN2+ was 34 % (38/111) for HPV DNA and 39 % (30/77) for the 5-type mRNA test. The PPVs for CIN3+ were 13.5 % (15/111) and 19.5 % (15/77) for the DNA and the 5-type mRNA test (Table 3). The increased referral rate to biopsy among DNA-tested women relative to mRNA-tested women resulted in 10 more cases of normal histology, 10 more cases of CIN1, and eight more cases of CIN2 (Table 4).

**Table 2:** Most severe histology from biopsy/cone specimen by HPV test

Histology	HPV DNA		5-type-mRNA	
	*N =* 105	% (95 % CI)	*N =* 77	% (95 % CI)
Normal/CIN1	67	63.8 (54.6–73.0)	47	61.0 (50.1–71.9)
CIN2	23	21.9 (14.0–29.8)	15	19.5 (10.7–28.3)
CIN3+	15	14.3 (7.6–21.0)	15	19.5 (10.7–28.3)

*CIN* cervical intraepitelial neoplasi

*HPV DNA* HPV DNA test (Cobas 4800)

*5-type-mRNA* HPV mRNA test (PreTect HPV-Proofer)

**Table 3:** Test performance of HPV DNA test (*N =* 504) and 5-type-mRNA test (*N =* 536) in solved cases

Triage status	CIN2+	CIN1-	Total
HPV DNA positive	38	73	111^a^
HPV DNA negative	0	393	393
Total	38	466	504
Triage status	CIN2+	CIN1-	Total
HPV mRNA positive	30	47	77
HPV mRNA negative	8	451	459
Total	38	498	536

*CIN2+* CIN2, CIN3, ACIS, and cervical cancer

*CIN1-* Normal and CIN1

^a^Of the 111 women with a positive HPV DNA test, six women had normal cytology and a negative HPV DNA test at second follow-up and returned to screening at 3-year interval

**Table 4:** HPV positivity, genotype, and HPV test by stage of triage and histology

	HPV DNA					5-type-mRNA				
		HPVNeg.	HPV 16	HPV 18	HPV other		HPVNeg.	HPV 16	HPV 18	HPV other
Stages of triage	N	%	%	%	%	N	%	%	%	%
At triage^a^	564	69.7	8.5	2.3	19.5	564	81.4	8.7	2.1	7.8
Recommended biopsy^b^	141		28.4	8.5	63.1	92		45.7	10.9	43.5
Had biopsy^c^	105		36.2	7.6	56.2	77		51.9	9.1	39.0
By histology	N		%	%	%	N		%	%	%
Normal/CIN1	67		26.9	7.5	65.7	47		44.7	8.5	46.8
CIN2	23		39.1	13.0	47.8	15		53.3	20.0	26.7
CIN3	14		71.4	0.0	28.6	14		71.4	0.0	28.6
Sq. cell carcinoma	1		0.0	0.0	100	1		0.0	0.0	100

*HPV DNA other* HPV type 31, 33, 35, 39, 45, 51, 52, 56, 58, 59, 66, and 68

*5-type-mRNA other* HPV type 31, 33, and 45

*CIN* cervical intraepitelial neoplasi

^a^The Norwegian cervical cancer screening program recommended triage 6 to 12 months after the index ASC-US/LSIL

^b^Women with either a positive HPV DNA or a positive HPV mRNA test are recommended biopsy

^c^In total 141 women were recommended colposcopy by the DNA test, and 105 (74 %) met for biopsy. Out of the 92 women scheduled for biopsy by the 5-type mRNA test, 77 (84 %) made a visit (p = 0.12)

At triage, 65 % (386/564) were negative in both tests (DNA-/mRNA-), while 29 % (165/564) and 19 % (105/564) were positive with the DNA or 5-type mRNA test, respectively. In total, 98 women were double positive (DNA+/mRNA+). Among the 73 HPV DNA positive and 5-type mRNA negative women (DNA+/mRNA-), 69 were positive for HPV types other than 16 and 18, two for HPV16, and two for HPV18. Among women with a negative HPV DNA test, seven tests were positive with the 5-type mRNA test (DNA-/mRNA+), four HPV16 and three HPV other than 16 and 18. Among the 53 women testing positive for HPV16 (DNA+ and/or mRNA+), 44 tested positive in both tests (DNA+/mRNA+), 48 tested positive for HPV DNA, and 49 for 5-type mRNA. Similar results for HPV18, 11 women out of 14 were double positive (DNA+/mRNA+), 13 tested positive for HPV DNA, while 12 tested positive for 5-type mRNA. The largest difference was for HPV other types than HPV16 and HPV18. Only 39 out of 116 tested positive in both tests (DNA+/mRNA+), 110 tested positive for DNA, and 44 tested positive for 5-type mRNA.

Table 4 summarizes HPV positivity, genotype, stage of triage and histology by HPV test. The DNA test detected 23 cases of CIN2, relative to 15 with the 5-type mRNA test. There was concordance between tests in eight cases of HPV16, three cases of HPV18, and three cases of HPV other than 16 and 18. One CIN2 case testing HPV16 in the DNA test tested HPV other than 16 and 18 in the 5-type mRNA test. The eight additional cases of CIN2 detected by the DNA test were all negative for HPV 16/18 and positive for other than 16 and 18: three in the age group 25–34 years and five in the 35–69 group. All cases with CIN3 (*n =* 14) were concordant for HPV type in both tests (*n =* 10 for HPV16, *n =* 4 for HPV other than 16 or 18).

In total 89 % (504/564) of the DNA cases were solved across the time frame of the study, relative to 95 % (536/564) of the 5-type mRNA-tested cases (*p <* 0.01). The cumulative proportions of cases solved within 12 and 36 months were significantly higher for 5-type mRNA-tested subjects, 92 % (95 % CI: 90–94) and 96 % (95 % CI: 94–98), than DNA-tested subjects, 85 % (95 % CI: 82–88) and 90 % (95 % CI: 87–93)).

The sensitivity among solved cases for CIN2+ of the HPV DNA test was 100 % (38/38) relative to 79 % (30/38, 95 % CI: 67–92) for the 5-type mRNA test. The corresponding estimates of specificity among solved cases were 84 % (393/466, 95 % CI: 81–88), and 91 % (451/498, 95 % CI: 88–93) (Table 3) (*p <* 0.01). In Tables 5, 6, 7 and 8 we provide data on triage cytology (ASC-US or LSIL) by HPV test for CIN2+ and CIN3 + .

**Table 5:** Test performance of HPV DNA test and 5-type HPV mRNA test in repeated ASC-US in solved cases versus CIN2+

Triage status	CIN2+	CIN1-	Total
HPV DNA positive	25	62	87
HPV DNA negative	0	28	28
Total	25	90	115
Triage status	CIN2+	CIN1-	Total
HPV mRNA positive	18	45	63
HPV mRNA negative	5	45	50
Total	23	90	113

*CIN2+* CIN2, CIN3, ACIS, and cervical cancer

*CIN1-* Normal and CIN1

**Table 6:** Test performance of HPV DNA test and 5-type HPV mRNA test in repeated ASC-US in solved cases versus CIN3+

Triage status	CIN3+	CIN2-	Total
HPV DNA positive	9	78	87
HPV DNA negative	0	28	28
Total	9	106	115
Triage status	CIN3+	CIN2-	Total
HPV mRNA positive	9	54	63
HPV mRNA negative	0	50	50
Total	9	104	113

*CIN2+* CIN2, CIN3, ACIS, and cervical cancer

*CIN1-* Normal and CIN1

**Table 7:** Test performance of HPV DNA test and 5-type HPV mRNA test in repeated LSIL in solved cases versus CIN2+

Triage status	CIN2+	CIN1-	Total
HPV DNA positive	13	31	44
HPV DNA negative	0	5	5
Total	13	36	49
Triage status	CIN2+	CIN1-	Total
HPV mRNA positive	12	17	29
HPV mRNA negative	1	19	20
Total	13	46	49

*CIN2+* CIN2, CIN3, ACIS, and cervical cancer

*CIN1-* Normal and CIN1

**Table 8:** Test performance of HPV DNA test and 5-type HPV mRNA test in repeated LSIL in solved cases versus CIN3+

Triage status	CIN3+	CIN2-	Total
HPV DNA positive	6	38	44
HPV DNA negative	0	5	5
Total	6	43	49
Triage status	CIN3+	CIN2-	Total
HPV mRNA positive	6	23	29
HPV mRNA negative	0	20	20
Total	6	43	49

*CIN2+* CIN2, CIN3, ACIS, and cervical cancer

*CIN1-* Normal and CIN1

## Discussion

Our study shows that the 5-type HPV mRNA test had significantly lower positivity rate (19 %) than the 14-type HPV DNA test (29 %), which led to a significantly higher referral rate to colposcopy for the HPV DNA test. Both tests diagnosed equal numbers of women with CIN3+, whereas the DNA test detected eight more cases of CIN2. All these CIN2 cases were of HPV types other than 16/18 in both tests, and they were negative for HPV mRNA 31, 33, and 45.

In agreement with other studies, the positivity rate of the HPV DNA test in triage of ASC-US/LSIL is nearly double compared to the 5-type HPV mRNA test (Table 9 [6, 7, 10–12, 16]). We found a 57 % higher referral rate using HPV DNA versus 5-type mRNA while others have reported a double referral rate ratio using HPV DNA compared to 5-type mRNA [6, 7, 10].

**Table 9:** Test-performance of the 5-type mRNA test and 13–14 types DNA tests in delayed triage and reflex testing of women with minor cytological lesions and CIN3+ as outcome

Ref.	Data collection	Year publ.	Country	Study design	Timing HPV test	Length f-up (mo)	Diagnosis	HPV test	N	N HPV positive	N Met for biopsy	CIN3+			
												Sens.	Spes.	PPV	NPV
6	July 2005– Dec. 2009	2013	Norway	Case-series	Delayed triage	≤36	Repeat ASCUS/LSIL	HC II 5 mRNA	2150 1543	1504 510	1 184 435	NR			
7	Jan. 2004– Dec. 2006	2011	Italy	Head-to-head	Reflex testing	≤2	ASCUS/ LSIL	HC II 5 mRNA	795 755	614 204	377 132	NR			
10	Jan. 2012– Sept. 2012	2014	Norway^a^	Head-to-head	Delayed triage	≤33	Repeat ASCUS/LSIL	COBAS 5 mRNA	281 281	92 37	65 26	100 75.0	77.8 91.6	6.2 11.5	100 99.6
11	Aug. 2005– Jan. 2007	2008	UK	Head-to-head	Reflex testing	Same day	≤ mild dyskaryosis	HC II 5 mRNA	567 558	NR	NRe	100 89.4	26.0 72.8	11.1 23.2	100 NR
12	Sept. 2007– Oct. 2009	2012	UK	Head-to-head	Reflex Testing	Same day	≤ mild dyskaryosis	HC II 5 mRNA	670 641	526 272	NRe	100 80.9	NR	9.2 16.6	100 NR
16	NR	2010	Canada	Head-to-head	Reflex testing	≤6	ASCUS/ LSIL	HC II 5 mRNA	781 781	619 328	NRe	NR			
A	Jan. 2012– Sep. 2013		Norway^a^	Head-to-head	Delayed testing	≤33	Repeat ASCUS/LSIL	COBAS 5 mRNA	564 564	171 105	105 77	100 100	80.0 85.2	13.9 16.3	100 100

A Present study

*Sens* Sensitivity; *Spes.* Specificity; *PPV* positive predictive value; *NPV* negative predictive value; *NR* Not reported; *NRe* Not relevant. All women had colposcopy regardless of HPV result

^a^Only solved cases are included in test-performance analysis

Most studies report a higher sensitivity for CIN2+ and CIN3+ using the HPV DNA test or 14-type HPV mRNA test (Hologic APTIMA) compared to the 5-type HPV mRNA test, whereas the specificity is significantly higher for 5-type HPV mRNA compared to HPV DNA (or 14- type HPV mRNA test) [6, 7, 16–18]. The higher specificity reflects the higher positive predictive value (PPV) for the HPV 5-type mRNA test relative to the HPV DNA test (or 14-type HPV mRNA test).

The major difference in test performance between the DNA and the 5-type mRNA test was HPV types other than 16/18, which were in most cases HPV mRNA-negative for 31/33/45. The choice of test is crucial to avoid over-diagnosis in triage of women with minor cytological lesions. Our data indicates that the difference in sensitivity/loss of CIN2 may be attributed to HPV types with a low oncogenic potential with slow progression into cancer. The next screening round will capture these women for follow-up/treatment if there is any progression. A triage HPV test with high specificity, targeting the HPV types with the highest potential for progression to cervical cancer, will reduce over-diagnosis and overtreatment, as observed in this study. Over-diagnosis is a cost-driver in unnecessary conizations and may lead to an increased risk of premature births and late abortions in subsequent pregnancies [19, 20] in this young population.

In our study the 5-type HPV mRNA test detected the same number of CIN3+, with a significant lower positivity rate and significant lower referral rate to colposcopy than the HPV DNA test. The risk of cervical cancer in women with ASC-US/LSIL is low and even lower if the HPV mRNA test is negative [6, 16, 21]. In Europe, HPV16 predominates in both CIN3 and cervical cancer. Other HPV types have a slower progression into cancer [22]. In countries with an organized cervical cancer screening program the risk of development of cervical cancer is higher for HPV types 16, 18, 31, 33 and 45 than for other HPV types [23]. These observations support the use of a specific HPV mRNA test detecting the five main HPV types in triage of women with minor cytological lesions.

In a meta-analysis of the accuracy of 5-type HPV mRNA tests, the pooled sensitivity for CIN2+ of the 10 included studies was 75 % and 76 % for the triage of ASC-US and LSIL, respectively [14]. It is well known that many cervical lesions with moderate or severe dysplasia will regress spontaneously. Only 5 % of women with CIN2 will develop cervical cancer without treatment [24]. Only 31 % of colposcopically visible lesions with CIN3 will progress to invasive cancer within 30 years [25]. About 40 % of CIN2 will regress within two years, and the regression rate of CIN2 caused by other HPV types than HPV type 16 is even higher [26]. It is probable that the 5-type HPV mRNA test in triage of women with minor cervical lesions identifies the majority of the lesions that are destined to progress to cancer [27, 28]. When women with ASC-US/LSIL and a negative 5-type mRNA test are returned to screening in three years, we can reduce overtreatment of women with CIN1-2 caused by HPV types with a low risk of progression [22, 23, 26, 29].

The experience in the Department of Pathology, Ålesund Hospital, is that the 5-type HPV mRNA test has a high specificity and a high positive predictive value. This makes it useful for triage of women with minor cervical lesions.

## Conclusions

5-type HPV mRNA is more specific than HPV DNA in triage of women with repeated ASC-US/LSIL. The referral rate for colposcopy after repeated ASC-US/LSIL was 57 % higher for DNA+ relative to mRNA+ cases, with the same detection rate of CIN3+. It is important to consider the trade-off between sensitivity and specificity of the diagnostic test when designing screening algorithms.

## Abbreviations

ASC-H, atypical squamous cells – cannot exclude HSIL; ASC-US, atypical squamous cells of undetermined significance; CIN, cervical intraepithelial neoplasia, also known as cervical dysplasia; CIN1, CIN2, CIN3, cervical intraepithelial neoplasia grade 1, 2 or 3, also known as low grade, moderate or severe cervical dysplasia; CIN2+, CIN2, CIN3, adenocarcinoma in situ (ACIS) or cervical cancer DNA: Deoxyribonucleic acid; HPV, human papillomavirus; HPV DNA test, cobas 4800 detects DNA from 14 high-risk HPV types (16, 18, 31, 33, 35, 39, 45, 51, 52, 56, 58, 59, 66 and 68) at clinically relevant infection levels; HPV mRNA test, PreTect HPV-Proofer detects E6/E7 mRNA of 5 HPV types (16, 18, 31, 33 and 45); HSIL, High grade squamous intraepithelial lesion; LBC, liquid-based cytology; LSIL, low grade squamous intraepithelial lesion; mRNA, messenger RNA; NPV, negative predictive value; Pap smear, the Papanicolaou test, also known as Pap test, cervical smear or cervical cytology; PPV, positive predictive value; RNA, ribonucleic acid; WHO, the World Health Organization
